# Comparison of Risk Assessment Strategies for Patients with Diabetes Mellitus and Stable Chest Pain: A Coronary Computed Tomography Angiography Study

**DOI:** 10.1155/2022/8183487

**Published:** 2022-01-25

**Authors:** Jia Zhao, Shuo Wang, Pengyu Zhao, Yong Huo, Chunjie Li, Jia Zhou

**Affiliations:** ^1^Department of Cardiology, Tianjin Chest Hospital, Tianjin, China; ^2^Graduate School, Tianjin Medical University, Tianjin, China; ^3^Graduate School, Tianjin University of Traditional Chinese Medicine, Tianjin, China; ^4^School of Electrical and Information Engineering, Tianjin University, Tianjin, China; ^5^Department of Cardiology, Peking University First Hospital, Beijing, China

## Abstract

**Background:**

To compare two risk assessment strategies to identify individuals likely to benefit from further imaging testing in patients with diabetes mellitus (DM) and stable chest pain (SCP) suspected of obstructive coronary artery disease (CAD).

**Methods:**

602 DM patients referred to coronary computed tomography angiography (CCTA) for SCP were included. They were divided into high- and low-risk groups according to the 2016 National Institute of Health and Care Excellence guideline-determined strategy (NICE strategy) which focused on symptom evaluation and 2019 European Society of Cardiology guideline-determined strategy (ESC strategy) which was based on pretest probability (PTP) sequentially determined by the ESC-PTP estimator and risk factor-weighted clinical likelihood (RF-CL) model, respectively. The associations of clinical outcomes with risk groups and net reclassification improvement (NRI) were evaluated.

**Results:**

The NICE and ESC strategy classified 44% and 39% patients into the low-risk group, respectively. Compared to the NICE strategy, the ESC strategy indicated stronger associations between risk groups and events (hazard ratios: 4.24 versus 1.91), intensive clinical management, and a positive NRI (27.71%, *p* < 0.0001). The application of the RF-CL model ameliorated the underestimation of risk in patients with borderline ESC-PTP, which principally account for the improvement of the ESC strategy.

**Conclusion:**

Compared to the NICE strategy, the ESC strategy seemed to be associated with greater efficiency in identifying high risk individuals in patients with DM and SCP.

## 1. Introduction

In patients with diabetes mellitus (DM), coronary artery disease (CAD) is a major cause of mortality [1]. Stable chest pain (SCP) is the most common clinical manifestation in patients with obstructive CAD. In an analysis for the largest contemporary cohort of SCP, the PROMISE trial, patients with DM were more likely to have a positive cardiovascular imaging testing (CIT) result and major adverse cardiovascular event (MACE) [2]. Consequently, a risk assessment strategy to efficiently identify high-risk individuals deriving maximum benefit from further CIT is initial and essential in the clinical management for DM patients presenting with SCP suggestive of obstructive CAD [2–4].

To improve this identification, the 2016 U.K. National Institute of Health and Care Excellence (NICE) guideline recommended a symptom-based risk assessment strategy for SCP [5]. However, this strategy has been controversial since its release [6–8] and numerous studies have indicated that atypical symptoms were more likely to be a manifestation in patients with DM [2, 3, 9]. On the other hand, the 2019 European Society of Cardiology (ESC) guideline advocated an updated pretest probability (PTP) estimator based on age, sex, and symptom and recommended CIT for patients with high ESC-PTP [10]. For patients with borderline ESC-PTP, the addition of other risk factors can improve the estimation of clinical likelihood of obstructive CAD [11].

The 2016 NICE guideline-determined risk assessment strategy (NICE strategy) [12, 13] and ESC-PTP estimator [14–16] have been externally validated in general SCP patients. But to date, no comparative analysis has been conducted to systematically evaluate the NICE strategy and 2019 ESC guideline-determined risk assessment strategy (ESC strategy) in patients with both DM and SCP, for whom the appropriate referral for intensive investigation was fundamental but difficult [2, 3, 17]. Thus, we aimed to compare the two newest risk assessment strategies to optimize decision-making of downstream clinical management in a coronary computed tomography angiography- (CCTA-) based cohort comprised of patients with DM and SCP.

## 2. Methods

### 2.1. Study Population

As described previously, 5289 patients referred to CCTA for SCP indicative of obstructive CAD were recruited from December 2015 to December 2017 in Tianjin Chest Hospital [18–21]. In the present analysis, 602 patients with a diagnosis of DM were included and followed up until December 2019. Patients were considered suffering from DM if one of the following was met: treatment with insulin or hypoglycemic medications, fasting blood glucose ≥ 7.0 mmol/L, a 2 h plasma glucose level on their oral glucose tolerance test ≥ 11.1 mmol/L, or a glycated hemoglobin value ≥ 6.5%. This observational study was conducted after obtaining the informed consent from the participating patients and upon the approval by the ethics committee of Tianjin Chest Hospital.

### 2.2. Baseline Data

Baseline data such as age, sex, hypertension, hyperlipidemia, smoking, abnormal electrocardiograph, creatinine, and symptom were collected and defined as described previously [18–21]. SCP symptom was categorized as nonanginal chest pain, atypical angina, or typical angina [22]. For each patient, creatinine was routinely measured unless the measurement has happened within 2 months before CCTA. The estimated glomerular filtration rate was calculated based on the CKD-EPI formula [23].

### 2.3. Risk Assessment Strategies

A patient in the high-risk group based on each strategy should take CIT. Details of risk groups in the NICE and ESC strategy were as follows [5, 10]:

NICE strategy: patients with nonanginal SCP and normal ECG were at low risk. The high-risk group included SCP patients who were diagnosed with typical and atypical angina or nonanginal pectoris with abnormal ECG [5].

ESC strategy: PTP of obstructive CAD was determined according to the ESC-PTP estimator based on age, sex, and symptom [10]. Patients with ESC‐PTP < 5% were divided into the low-risk group, and patients with ESC‐PTP > 15% were divided into the high-risk group. For other patients, we used the risk factor-weighted clinical likelihood (RF-CL) model for further assessment [24]. The RF-CL model incorporating clinical variables plus age, sex, and symptom showed the most robust performance. According to the data from the original study of the RF-CL model, low RF-CL (<15%) was associated with less obstructive CAD (<5%) and risk of clinical events (<2% annual risk) [24]. Thus, patients with ESC-PTP between 5% and 15% and RF‐CL < 15% were at low risk, and patients with ESC-PTP between 5% and 15% and RF‐CL > 15% were at high risk.

### 2.4. CCTA

All scans were performed according to the established guideline [25] and institutional protocols [18–21]. In image evaluation, each coronary segment with a >2 mm diameter was analyzed for the presence of coronary diameter stenosis. According to the Coronary Artery Disease-Reporting and Data System [26], the maximal degree of coronary diameter stenosis was defined as 0%, 1-49%, and 50%. Obstructive CAD was defined as present if a patient had at least one lesion with ≥50% diameter stenosis or any unassessable segments at CCTA. The patient with obstructive CAD was defined as positive.

### 2.5. Follow-Up and Clinical Events

After CCTA, all patients were followed at 6, 12, 24, 36, and 48 months by phone call or physician visit. MACE, defined as cardiac death and myocardial infarction, was the primary endpoint. Cardiac death was defined as any death caused by cardiac disease or for which no other cause could be found. Myocardial infarction was defined as described in the Fourth Universal Definition of Myocardial Infarction [27]. The changes of downstream clinical management, which included medication prescriptions (such as antiplatelet agents, anti-ischemic drugs, and lipid-lowering agents), referrals to CIT (noninvasive and invasive imaging testing), and coronary revascularization (CR) within 60 days after CCTA, were identified on an electronic medical system. Increase of medication (IM), invasive coronary angiography (ICA), and CR were regarded as secondary endpoints. All endpoints were adjudicated via review of follow-up information and medical records by an independent clinical event committee who were blinded to other data.

### 2.6. Statistical Analysis

Student's *t*-test or Mann-Whitney *U* test was used to evaluate the differences in continuous variables appropriately. The *χ*^2^ test or Fisher exact test was used to evaluate the differences in categorical variables appropriately. All statistical analyses were performed using MedCalc (version 15.2.2; MedCalc Software, Mariakerke, Belgium) and R (version 3.2.4; R Foundation for Statistical Computing, Vienna, Austria). The discrimination and calibration of the ESC-PTP estimator were assessed by the area under receiver operating characteristic curve (AUC) and Hosmer–Lemeshow goodness-of-fit statistic (H-L *χ*^2^) according to the Transparent Reporting of a Multivariable Prediction Model for Individual Prognosis or Diagnosis (TRIPOD) statement [28]. Net reclassification improvement (NRI) was assessed in a reclassification table and used to determine how a risk assessment strategy reclassified patients into various risk groups compared with another [29]. The cumulative MACE-free survivals were estimated using Kaplan–Meier curves and were compared by the log-rank test. We used Cox proportional hazards regression models to calculate hazard ratios (HRs) and 95% confidence intervals (CIs). Two-tailed *p* < 0.05 was considered statistically significant.

## 3. Results

### 3.1. Baseline Characteristics

The study cohort consisted of 602 DM patients, of whom 45.02% (271/602) were found to have obstructive CAD on CCTA which are listed in Table 1. Most baseline characteristics were significantly associated with the presence of obstructive CAD. According to the NICE strategy, of the 602 patients, 43.68% (263/602) were assigned to the low-risk group. There were 87 patients with a RF‐CL < 15% among 208 patients with an ESC-PTP of 5-15%. Together with the 150 patients with an ESC-PTP below 5%, the ESC strategy totally classified 39.37% (237/602) into the low-risk group.

Table 2 shows the distribution of clinical characteristics by risk groups based on different strategies. Except hypertension and hyperlipidemia, differences of the other baseline characteristics were statistically significant between two risk groups based on the NICE strategy. There were significant differences in most characteristics except abnormal ECG in terms of the ESC strategy. Compared with low-risk patients, high-risk patients had more obstructive CAD (NICE strategy: 61% versus 25%, *p* < 0.0001; ESC strategy: 71% versus 5%, *p* < 0.0001) and MACE (NICE strategy: 9% versus 5%, *p* = 0.0422; ESC strategy: 10% versus 3%, *p* = 0.0001).

### 3.2. Follow-Up

**Figure 1:**
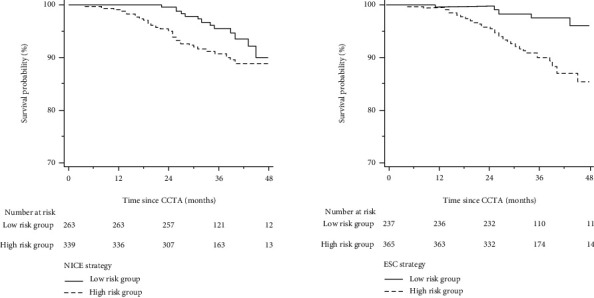
Cumulative survival probability from MACE in low- and high-risk groups determined by the NICE and ESC strategy. Abbreviations as in Table 3.

Patients were followed up for a median of 36 (interquartile range: 30 to 43) months, and 37 patients experienced MACE (8 cardiac deaths and 29 nonfatal MI).  Figure 1 illustrates the Kaplan–Meier estimates of patients surviving free from MACE. The high-risk group according to both NICE and ESC strategies had a significantly higher risk of MACE, respectively (*p* for the log-rank test: 0.0445 for the NICE strategy and 0.0003 for the ESC strategy), but the association of ESC strategy-determined risk groups (high versus low) with MACE was stronger than that of the NICE strategy (HR for NICE strategy: 1.91, 95% CI 1.01-3.63, *p* = 0.0485; HR for ESC strategy: 4.24, 95% CI 1.80-9.97, *p* = 0.0010).

### 3.3. Subsequent Clinical Management

**Figure 2:**
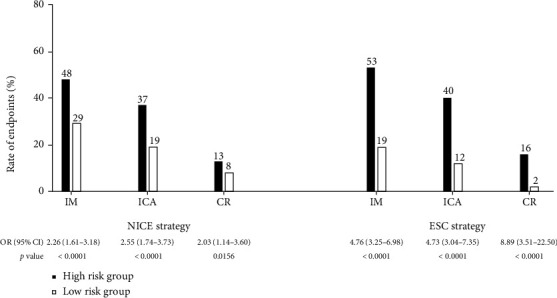
Rates for secondary endpoints in low- and high-risk groups determined by the NICE and ESC strategy. ICA: invasive coronary angiography; IM: increase of medication; CR: coronary revascularization; other abbreviations as in Table 3.

The associations between risk groups and secondary endpoints according to the NICE and ESC strategy are manifested in Figure 2. 175 patients had ICA based on CCTA, 138 patients had obstructive CAD on ICA, and 65 patients underwent CR. Compared with low-risk patients, high-risk patients had more IM (NICE strategy: 48% (164/339) versus 29% (77/263), odds ratio (OR): 2.26, 95% CI: 1.61-3.18, *p* < 0.0001; ESC strategy: 53% (195/365) versus 19% (46/237), OR: 4.76, 95% CI: 3.25-6.98, *p* < 0.0001), ICA (NICE strategy: 37% (125/339) versus 19% (49/263), OR: 2.55, 95% CI: 1.74-3.73, *p* < 0.0001; ESC strategy: 40% (145/365) versus 12% (29/237), OR: 4.73, 95% CI: 3.04-7.35, *p* < 0.0001), and CR (NICE strategy: 13% (44/339) versus 8% (20/263), OR: 2.03, 95% CI: 1.14-3.60, *p* = 0.0156; ESC strategy: 16% (59/365) versus 2% (5/237), OR: 8.89, 95% CI: 3.51-22.50, *p* < 0.0001).

### 3.4. Validation of the ESC-PTP Estimator

**Figure 3:**
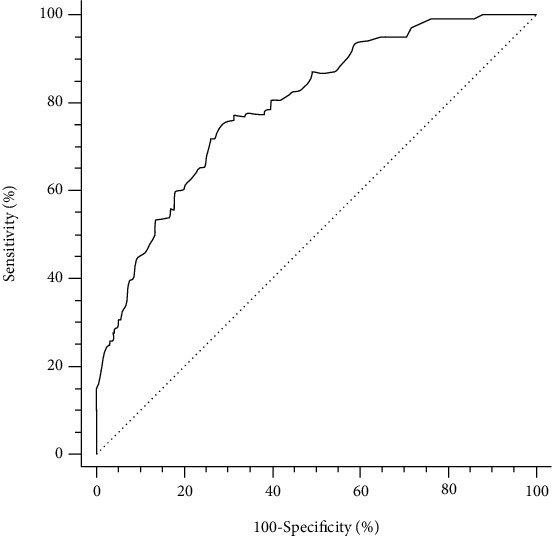
ROC curves for the ESC-PTP estimator to predict obstructive CAD. ROC: receiver operating characteristic; other abbreviations as in Table 4.

**Figure 4:**
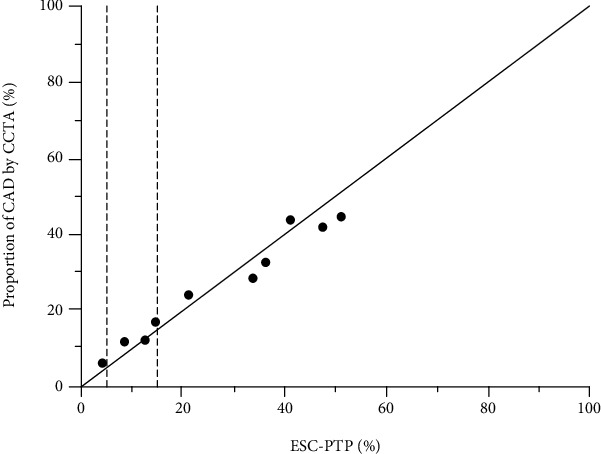
Comparison of ESC-PTP and proportion of obstructive CAD on CCTA by deciles of ESC-PTP. The area between two dotted lines represents ESC-PTP between 5% and 15%. CCTA: coronary computed tomographic angiography. Other abbreviations as in Figure 3.

The receiver operating characteristic curves of the ESC-PTP estimator are illustrated in Figure 3. The discrimination of the ESC-PTP estimator was acceptable, with an AUC of 0.783 (95% CI 0.747 to 0.819, *p* < 0.0001). The calibration plot for the ESC-PTP estimator is presented in Figure 4. Graphically, the ESC-PTP estimator underestimated the probability of obstructive CAD in patients with an ESC-PTP between 5% and 15% and overestimated the probability of obstructive CAD in patients with an ESC‐PTP > 15%, resulting in a poor calibration (H-L *χ*^2^ = 92.47, *p* < 0.0001).

### 3.5. Comparison of the ESC Strategy and NICE Strategy by NRI

Table 3 is the reclassification table comparing the ESC strategy to the NICE strategy. Compared to the NICE strategy, among the 331 negative patients, 35 patients were correctly reclassified from high- to low-risk groups by the ESC strategy, but 8 negative patients were incorrectly reclassified from low- to high-risk groups by the ESC strategy. Among the 271 positive patients, the ESC strategy correctly reclassified 59 patients from low- to high-risk groups, but 6 patients were incorrectly reclassified from high- to low-risk groups. Therefore, the NRI of the ESC strategy compared with the NICE strategy was 8.15% for negative patients, 19.66% for positive patients, and 27.71% for all patients.  Table 4 is the reclassification table comparing the RF-CL model to the NICE strategy in patients with ESC-PTP between 5% and 15%. Compared to the NICE strategy, the RF-CL model correctly reclassified 36 positive patients into the high-risk group, in large measure accounting for the NRI of 32.29% in positive and the NRI of 42.11% in all. As shown in Table 5, the improvement was attenuated when the analysis was applied to patients with ESC-PTP below 5% and above 15%, either comparing the RF-CL model to the NICE strategy (NRI = 24.33%, *p* < 0.0001) or comparing the ESC strategy to the NICE strategy (NRI = 19.88%, *p* < 0.0001), resulting from the similar classification of the ESC strategy and RF-CL model (NRI = −4.45%, *p* = 0.0598).

## 4. Discussion

In this CCTA-based cohort comprised of patients with DM and SCP, based on two newest risk assessment strategies, low-risk groups were associated with fewer obstructive CAD, MACE, and clinical interventions than high-risk groups did. Compared to the NICE strategy which focused on symptom evaluation, the ESC strategy which sequentially incorporated the ESC-PTP estimator with the RF-CL model had more potential to optimize decision-making of downstream referral for CIT in patients with DM and SCP.

It has been well established that DM confers a two-fold increased risk of MACE in patients presenting with SCP potentially related to CAD [2]. Thus, the referral of CIT to screening for obstructive CAD guided by the risk assessment strategy is vital in the clinical management of patients with DM and SCP, but the most efficient strategy for these patients has been debated until now [2, 3]. In stark contrast to the “screen all” strategy which was not supported by contemporary evidence [17, 30], the NICE strategy recommended a simple evaluation based on symptom [5] and improved diagnostic certainty and clinical outcomes compared to traditional PTP calculator-based strategies in two external validation studies conducted in general SCP patients [12, 13]. However, compared to the ESC strategy, the NICE strategy was demonstrated to be less efficient in the risk assessment for patients with DM and SCP.

Because of autonomic neuropathy affecting the pain perceptual threshold, the association between ischemia and SCP may be diminished, which could lead to an atypical presentation in patients with DM [9, 31, 32]. As a result, current evidence implied that the suboptimal performance of the NICE strategy may, to a large extent, be attributed to the insufficient power of symptom evaluation alone in patients with DM and SCP. In the DM subgroup of the PROMISE cohort, neither typical nor atypical chest pain was an independent predictor of positive CIT [2]. Meanwhile, an analysis conducted in 8662 patients referred for new-onset SCP suggested that among patients diagnosed with noncardiac chest pain, those with DM remained at two-fold increased risk of MACE, compared with non-DM patients [3]. In conformity with these findings, when comparing the NICE strategy to the ESC strategy in the present study, the NRI was negative and the association between study endpoints and risk groups was attenuated.

Both the ESC-PTP estimator and RF-CL model were developed in the most contemporary SCP cohorts and indicative of the best performance to predict obstructive CAD and MACE in general SCP patients [14–16, 24], which were compliant with the modest AUC for the ESC-PTP estimator and positive NRI comparing the RF-CL model to the NICE strategy in the present study. In addition, the RF-CL model has also taken the interaction effect between symptom and DM into account [24]. As a result, the ESC strategy based on the sequential amalgamation of the ESC-PTP estimator and RF-CL model demonstrated superiority in terms of the diagnosis for obstructive CAD, prediction of MACE, and use of downstream diagnostic and therapeutic interventions in patients with DM and SCP.

As illustrated in Figure 3, the ESC-PTP estimator overestimated the probability of obstructive CAD in patients with ESC‐PTP > 15%. The overestimation may not change the further clinical management, because all patients with ESC‐PTP > 15% should be referred to CIT according to the ESC strategy [11]. On the contrary, the underestimation for probability of obstructive CAD in patients with ESC-PTP between 5% and 15% may result in a significant number of missing referrals for CIT. To provide a more in-depth and comprehensive insight into the risk assessment for patients with borderline ESC-PTP, we also conducted the comparison between the RF-CL model and NICE strategy in patients with ESC-PTP between 5% and 15% (Table 4) and among the RF-CL model, ESC strategy, and NICE strategy in ESC-PTP below 5% and above 15% (Table 5), respectively. Taking all these into consideration, when comparing the ESC strategy to the NICE strategy, the application of the RF-CL model obviously ameliorated the underestimation of risk in 208 patients with borderline ESC-PTP and the total reclassification of 59 positive patients should be principally (61.02%, 36/59) attributed to this application.

More importantly, the RF-CL model incorporated symptom assessment plus risk factors which were easily accessible in daily clinical practice. Thus, one additional collection of information for risk factors in every 602/208 ≈ 3 DM patients and one avoidance of missing referrals for CIT corresponding to 208/(36‐5) ≈ 7 additional collection of information for risk factors made the ESC strategy more efficient. As mentioned above, the optimal risk assessment strategy to guide the screening of CAD in patients with DM and SCP has great clinical importance. In this context, instead of the NICE strategy which mainly focused on symptom evaluation, the ESC strategy which sequentially incorporated different PTP models might provide more feasible identification of DM patients who may derive maximal benefit from further CIT.

### 4.1. Limitations

Although this is the first study to evaluate proposed risk assessment strategies for patients with DM and SCP, several issues merit consideration. First, this study was an observational cohort. Clinical management of patients with DM and SCP before and after CCTA relied on a local physician. More details about medical therapy and CR during follow-up were not available. Thus, whether the ESC strategy will lead to more appropriate decision-making of downstream referral and better clinical outcomes for patients with DM and SCP needs to be addressed in further studies, such as randomized controlled trials. Second, using data from the PROMISE cohort, Fordyce et al. developed a new tool to identify patients deriving minimal value from CIT [33]. Although the PROMISE minimal risk tool [34] has been externally validated, no recent clinical guideline recommends it as the risk assessment tool for patients with SCP. Third, this analysis focused on the presence of obstructive CAD documented by CCTA. Previous studies have demonstrated that CCTA had a high negative predictive value compared with ICA [35, 36]. So CCTA could offer robust reassurance for both strategies to exclude obstructive CAD. Moreover, we defined unassessable segments as positive ones based on current guideline recommendations in which further testing should be referred for nonconclusive CCTA. Fourth, a coronary artery calcium score [20, 37] and high-sensitivity cardiac troponin [38, 39] have shown the potential to improve risk assessment for patients with DM and SCP. However, additional imaging or blood testing is needed for the two attractive biomarkers, and their cost-effectiveness warrants further evaluation. Fifth, as the majority of patients had missing data about other ECG changes such as *Q* wave, we only analyzed ST-T changes. This could reduce the size of the high-risk group in the NICE strategy, especially in DM patients for whom the silent ischemia is common [9, 31, 32]. Finally, more researches about the performance of different strategies in different subgroups of age and sex were needed in the future.

## 5. Conclusions

Compared to the symptom-focused strategy, the ESC strategy based on PTP estimation seemed to be associated with greater efficiency in identifying high-risk individuals who may derive maximum benefit from further CIT in patients with DM and SCP. This superiority should be dominantly ascribed to the application of the RF-CL model in borderline patients. For more accurate and convenient risk assessment in patients with DM and SCP suggestive of obstructive CAD, further investigations with comprehensive and rigorous design are needed.

## Figures and Tables

**Table 1:** Baseline characteristics by the presence of obstructive CAD on CCTA.

Characteristic	Total	Obstructive CAD		*p*
	*N* = 602	Yes (*N* = 271)	No (*N* = 331)	
Age (years, mean ± SD)	62.26 ± 11.61	65.83 ± 12.79	59.34 ± 11.96	<0.0001
Male	331 (55)	176 (65)	155 (47)	<0.0001
Hypertension	409 (68)	198 (73)	211 (64)	0.0188
Hyperlipidemia	313 (52)	160 (59)	153 (46)	0.0023
Smoking	284 (47)	143 (53)	141 (43)	0.0162
Abnormal ECG	259 (43)	132 (49)	127 (38)	0.0136
eGFR (mL/min/1.73 m^2^, mean ± SD)	71.59 ± 9.47	70.34 ± 10.49	72.61 ± 12.07	0.0152
Symptom				0.0182
Nonanginal chest pain	284 (47)	115 (42)	169 (51)	
Atypical angina	239 (40)	110 (41)	129 (39)	
Typical angina	79 (13)	46 (17)	33 (10)	

SD: standard deviation; CAD: coronary artery disease; ECG: electrocardiogram; CCTA: coronary computed tomographic angiography; eGFR: estimated glomerular filtration rate. Values are presented as *n* (%) unless stated otherwise.

**Table 2:** Characteristics by risk groups based on the NICE and ESC strategy.

	Total	NICE strategy			ESC strategy		
		Low	High	*p*	Low	High	*p*
	*n* = 602	*n* = 263	*n* = 339		*n* = 237	*n* = 365	
Age (years, mean ± SD)	62.26 ± 11.61	59.99 ± 12.53	64.02 ± 12.10	<0.0001	57.44 ± 12.44	65.39 ± 12.27	<0.0001
Female	331 (55)	128 (49)	203 (60)	0.0078	101 (43)	230 (63)	<0.0001
Hypertension	409 (68)	172 (65)	237 (70)	0.2763	143 (60)	266 (73)	0.0017
Hyperlipidemia	313 (52)	130 (49)	183 (54)	0.3045	109 (46)	204 (56)	0.0219
Smoking	284 (47)	111 (42)	173 (51)	0.0385	91 (38)	193 (53)	0.0007
Abnormal ECG	259 (43)	0 (0)	259 (76)	<0.0001	95 (40)	164 (45)	0.2760
Symptom				<0.0001			0.0001
Nonanginal chest pain	284 (47)	263 (100)	21 (6)		91 (38)	193 (53)	
Atypical angina	239 (40)	0 (0)	239 (71)		100 (42)	139 (38)	
Typical angina	79 (13)	0 (0)	79 (23)		46 (20)	33 (9)	
Obstructive CAD^b^	271 (45)	65 (25)	206 (61)	<0.0001	12 (5)	259 (71)	<0.0001
MACE	45 (7)	13 (5)	32 (9)	0.0422	6 (3)	39 (10)	0.0001
Cardiac death	11 (2)	2 (1)	9 (3)	0.1244	0 (0)	11 (3)	0.0044
Nonfatal MI	34 (5)	11 (4)	23 (6)	0.2325	6 (3)	28 (7)	0.0067

SD: standard deviation; CAD: coronary artery disease; NICE strategy: 2016 National Institute of Health and Care Excellence guideline-determined risk assessment strategy; ESC strategy: 2019 European Society of Cardiology guideline-determined risk assessment strategy; ECG: electrocardiogram; MI: myocardial infarction; MACE: major adverse cardiovascular events. Values are presented as *n* (%) unless stated otherwise.

**Table 3:** Reclassification table comparing the ESC strategy to the NICE strategy.

	Risk groups by ESC strategy		Total	Reclassification		NRI	*p*
	Low	High		Up	Down		
Risk groups by NICE strategy							
Negative patients				2.42%	10.57%	27.71%	<0.0001
Low	190	8	198				
High	35	98	133				
Total	225	106	331				
Positive patients				21.77%	2.21%		
Low	6	59	65				
High	6	200	206				
Total	12	259	271				

CAD: coronary artery disease; NICE strategy: 2016 National Institute of Health and Care Excellence guideline-determined risk assessment strategy; ESC strategy: 2019 European Society of Cardiology guideline-determined risk assessment strategy.

**Table 4:** Reclassification table comparing the RF-CL model to the NICE strategy in patients with borderline ESC-PTP.

	Risk groups by RF-CL model		Total	Reclassification		NRI	*p*
	Low	High		Up	Down		
Risk groups by NICE strategy							
Negative patients				2.68%	12.50%	42.11%	<0.0001
Low	65	3	68				
High	14	30	44				
Total	79	33	112				
Positive patients				37.50%	5.21%		
Low	3	36	39				
High	5	52	57				
Total	8	88	96				

RF-CL: risk factor-weighted clinical likelihood; ESC-PTP: 2019 European Society of Cardiology guideline-determined pretest probability; other abbreviations as in Table 3.

**Table 5:** Reclassification table comparing the RF-CL model, NICE strategy, and ESC strategy in patients with ESC-PTP below 5% and above 15%.

	Low	High	Total	Reclassification^∗^		NRI^†^	*p*
				Up	Down		
	Risk groups by ESC strategy						
Risk groups by NICE strategy							
Negative patients				2.28%	9.59%	19.88%	<0.0001
Low	125	5	130				
High	21	68	89				
Total	146	73	219				
Positive patients				13.14%	0.57%		
Low	3	23	26				
High	1	148	149				
Total	4	171	175				
	Risk groups by RF-CL model						
Risk groups by NICE strategy							
Negative patients				2.28%	14.61%	24.33%	<0.0001
Low	125	5	130				
High	32	57	89				
Total	157	62	219				
Positive patients				12.57%	0.57%		
Low	4	22	26				
High	1	148	149				
Total	5	170	175				
	Risk groups by ESC strategy						
Risk groups by RF-CL model							
Negative patients				6.85%	1.83%	-4.45%	0.0598
Low	142	15	157				
High	4	58	62				
Total	146	73	219				
Positive patients				1.71%	1.14%		
Low	2	3	5				
High	2	168	170				
Total	4	171	175				

Abbreviations as in Table 3.

## Data Availability

The data used to support the findings of this study are available from the corresponding author upon request.
